# Eye movements of patients with schizophrenia in a natural environment

**DOI:** 10.1007/s00406-014-0567-8

**Published:** 2014-12-04

**Authors:** Stefan Dowiasch, Bianca Backasch, Wolfgang Einhäuser, Dirk Leube, Tilo Kircher, Frank Bremmer

**Affiliations:** 1 Department of Neurophysics, Philipps-University Marburg, Karl-von-Frisch-Straße 8a, 35043 Marburg, Germany; 2 Department of Psychiatry and Psychotherapy, Philipps-University Marburg, Rudolf-Bultmann-Straße 8, 35039 Marburg, Germany; 3 AWO Centre of Psychiatry Halle, Clinic for Psychiatry and Psychotherapy, Zscherbener Str. 11, 06124 Halle, Germany

**Keywords:** Eye movements, Real-world gaze, Schizophrenia, Natural environment, Self-motion

## Abstract

Alterations of eye movements in schizophrenia patients have been widely described for laboratory settings. For example, gain during smooth tracking is reduced, and fixation patterns differ between patients and healthy controls. The question remains, whether such results are related to the specifics of the experimental environment, or whether they transfer to natural settings. Twenty ICD-10 diagnosed schizophrenia patients and 20 healthy age-matched controls participated in the study, each performing four different oculomotor tasks corresponding to natural everyday behavior in an indoor environment: (I) fixating stationary targets, (II) sitting in a hallway with free gaze, (III) walking down the hallway, and (IV) visually tracking a target on the floor while walking straight-ahead. In all conditions, eye movements were continuously recorded binocularly by a mobile lightweight eye tracker (EyeSeeCam). When patients looked at predefined targets, they showed more fixations with reduced durations than controls. The opposite was true when participants were sitting in a hallway with free gaze. During visual tracking, patients showed a significantly greater root-mean-square error (representing the mean deviation from optimal) of retinal target velocity. Different from previous results on smooth-pursuit eye movements obtained in laboratory settings, no such difference was found for velocity gain. Taken together, we have identified significant differences in fundamental oculomotor parameters between schizophrenia patients and healthy controls during natural behavior in a real environment. Moreover, our data provide evidence that in natural settings, patients overcome some impairments, which might be present only in laboratory studies, by as of now unknown compensatory mechanisms or strategies.

## Introduction

Eye movements in schizophrenia patients have been a topic of research for more than a century [1]. A large number of different eye-movement abnormalities have been described. Examples include decreased smooth-pursuit gain [2–4], increased anti-saccade error rates and latencies [5], changes in saccade dynamics [6, 7], or different fixation patterns when viewing static pictures [8, 9].

Except one recently published study that found differences in scan-path patterns in unfamiliar environments between schizophrenia patients and healthy controls [10], previous work on eye-movement abnormalities in schizophrenia has been performed in laboratory settings. Recent studies in healthy participants have documented differences in eye-movement behavior measured in the laboratory as compared to real-life scenarios [11, 12]. Further, a real-life gaze-tracking study on neurological patients (patients with either idiopathic Parkinson’s disease or progressive supranuclear palsy [13]) has demonstrated that mobile eye tracking in natural environments offers a simple, rapid, and reliable tool with good acceptance by the patients. Additionally, it showed results partially distinct from laboratory measurements. Accordingly, we aimed at investigating how well eye-movement abnormalities found in schizophrenia patients in laboratory studies transfer to real-world settings. That is, we asked whether some of the differences between schizophrenia patients and healthy controls could be attributed, at least partly, to the laboratory measurement settings. These typically constrain movements (e.g., by restraining the head) and focus on single isolated aspects, whereas real-world tasks usually induce interaction of multiple sensory and eye-movement systems. Differences between laboratory data as reported in the literature and real-world scenarios could point toward compensatory mechanisms in oculomotor control in schizophrenia, which are only available during natural vision. Moreover, the simplicity of modern mobile eye trackers offers the opportunity to be used as a tool in the daily clinical routine. Importantly, even if we find differences, this will not invalidate laboratory eye-movement experiments. To the contrary, we consider laboratory and real-world measurements complementary, in that they shed light on the question how oculomotor deficiencies as measured in the laboratory manifest in patients’ activities of daily living.

Active visual tracking of objects during self-motion is a common behavior during everyday life [14]. However, self-motion through an environment induces one of the most fundamental causes for differences between eye movements in the laboratory and the real world. During walking, the eye-movement system encounters distinct demands as compared to sitting still in the laboratory, which is reflected in qualitatively different oculomotor behavior [15, 16]. For example, while tracking movements as performed in the laboratory are typically restricted to eye movements, tracking behavior in the real world is usually accompanied by head movements and vestibular–ocular reflexes. Therefore, real-world oculomotor function faces the additional challenge to integrate self-motion information in order to operate optimally. Yet, in turn, oculomotor deficits may also be compensated by other effectors. The usually required smooth eye movements during visual tracking of a target are impaired in schizophrenia patients when tested in laboratory settings. At the cortical level, the processing of self-motion signals takes place primarily in the dorsal visual pathway [17]. Due to impairments in the magnocellular pathway, this part of the visual cortical system is suggested to be dysfunctional in schizophrenia [18, 19]. Indeed, some motion sensitive areas seem to be impaired in schizophrenia patients [20, 21]. Hence, we hypothesized that the processing of self-motion information, which in humans as well as non-human primates (NHPs) is explicitly encoded in areas like the ventral intraparietal area (VIP [22–25]) and the medial superior temporal area (MST [26–28]), is dysfunctional in schizophrenia patients when performing activities of daily living.

In the current study, we measured eye-movement parameters in schizophrenia patients and healthy controls using a mobile lightweight eye tracker, the EyeSeeCam (ESC) [29]. The ESC allows examining eye movements with no physical restrictions in a natural setting during simple tasks like walking. We aimed to find differences between schizophrenia patients and healthy controls in fundamental eye-movement parameters such as fixation duration and saccade amplitude as well as impairments in smooth tracking eye movements. Such differences may interact with task demands or depend on the additional sensory information available in natural settings as compared to the laboratory. If so, this would suggest that schizophrenia patients can compensate for specific deficits under certain behavioral or environmental conditions. If true, it would underline the need for addressing real-world situations to complement laboratory measurements toward a full understanding of the mechanisms underlying oculomotor dysfunctions in patients with schizophrenia.

## Materials and methods

Twenty schizophrenia patients (ICD 10: F20.0) and twenty healthy age- and sex-matched controls participated in the study. All of them gave their written informed consent. The study was approved by the local ethics committee and was in accordance with the Declaration of Helsinki. In this study, we focused solely on the paranoid subtype of schizophrenia to minimize variability of test results due to the heterogeneity of this disease. This choice guaranteed to cover the most prevalent subtype. The patients were recruited at the Department of Psychiatry and Psychotherapy at the University of Marburg. For more details on the patient and control groups, see Table 1.

**Table 1:** Overview of demographic and clinical values of patients and healthy controls

	Patients mean (SD)	Healthy controls mean (SD)	*p* (*T* test)
Age (years)	31.3 (9.4)	33.2 (6.8)	0.48
Sex	M: 17/w: 3	m: 18/w: 2	0.64
In-/outpatient	In: 9/out: 11		
Age of onset (years)^a^	25.8 (4.7)		
Duration of illness (years)	6.7 (7.4)		
No. of episodes	3.9 (3.2)		
CPZ equivalent dose (mg/day)	865 (789)		
PANSS score [66]	65.6 (23.0)		

^a^Age of onset could not be determined precisely in 4 patients

All patients were on neuroleptic medication. Some patients also were administered antidepressants (*n* = 6) and anticholinergics (*n* = 3). Participants were excluded if they had a history of serious head injury, general medical, or neurological disease. All participants had normal or corrected-to-normal vision. In addition, healthy controls were excluded if they or a first-degree relative had a psychiatric disorder. All participants were instructed by an experienced researcher and were able to perform the tasks.

We used a mobile lightweight eye tracker, the EyeSeeCam (ESC), to study binocular eye movements with a sampling rate of 280 Hz, a spatial resolution of 0.02°, and a precision of about 0.1° [30]. Although this does not compare to state-of-the-art laboratory equipment, these values compare well to other mobile eye-tracking systems used in natural environments and allowed us to determine saccadic eye-movement parameters reliably. The system was calibrated before each measurement by matching the gaze direction of each subject with the position of 5 predefined targets, projected with a head-fixed laser pointer to a plain wall in two meter distance. The mean error threshold for a successful calibration was set to 0.5°. Participants were asked to perform different tasks in an indoor environment and were instructed to act as they normally would throughout these tasks.

**Fig. 1:**
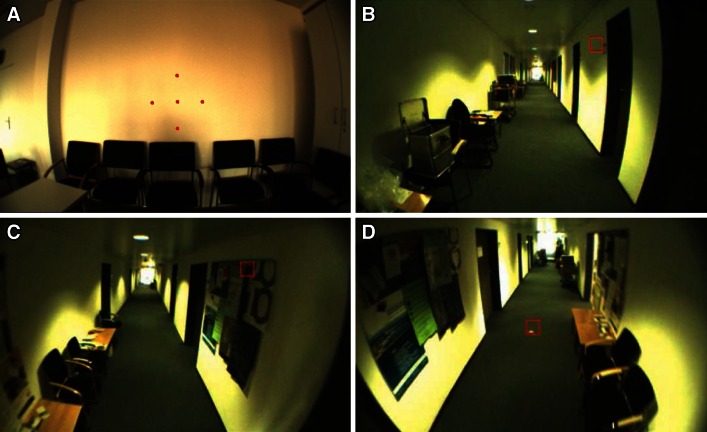
Illustration of a typical scene during each of four different tasks. Images were taken from the head-mounted camera of the ESC. The *red square* indicates the current gaze position of a participant. **a** Task I: fixating stationary targets with a fixed distance of 7° in a freely selectable and self-paced order as projected by a head-fixed laser pointer of the ESC (enhanced in this figure for visualization). **b** Task II: sitting in a clinic hallway with free gaze. **c** Task III: walking down the hallway with free gaze and **d** Task IV: visually tracking two stationary targets on the floor while walking straight-ahead

The paradigm consisted of four different tasks, which are illustrated schematically in Fig. 1. In short, during task I, subjects had to successively fixate predefined targets in a self-chosen order. Afterward, subjects sat in a hallway with free gaze (task II) or walked along that hallway with no additional task (task III). Finally, in task IV, subjects were asked to visually track a fixed spot on the ground while walking toward it. Across the group of patients and controls, the duration of the full set of measurements, i.e., performing all four tasks once including setup and calibration of the eye tracker, ranged from 5 to 10 min.

Video sequences of self-motion and eye position data were analyzed offline using MATLAB 2010b (the MathWorks, Inc., Natick, Massachusetts, USA).

Initially, the raw eye position data recorded with the ESC were analyzed for blinks and other artifacts due to reflections by external light sources. Blinks were identified as the absence of more than 5 samples (18 ms), and eye traces were cleaned for blink artifacts such that 8 samples (29 ms) before the start of a blink and 12 samples (43 ms) after a blink were not considered for further analysis. For the whole measurement, this resulted in an overall rejection of 2.9 % of all recorded samples from further analysis.

Afterward, horizontal and vertical eye speed was computed by the point-wise derivative of the respective eye-position values. Absolute eye velocities were calculated by taking the square root of the sum of the squared horizontal and vertical eye speed components.

Eye movements were classified as saccades if eye velocity was higher than 100°/s for at least 3 consecutive samples and if the eyes moved more than 0.5° in this period. In addition, a main-sequence analysis (peak velocity/amplitude) of all fast eye movements was performed by computing the power function fit ($v_{\text{peak}} = K*{\text{amplitude}}^{L}$) for eye position data from each subject and its corresponding 95 % confidence interval [31]. The remaining 5 % of all potential saccades outside this interval were classified as outliers and were not considered for further analysis. Oculomotor behaviors during which the eye position remained within a 1° - wide window in the horizontal and vertical direction for at least 150 ms were considered fixations.

To evaluate tracking behavior in tasks III and IV, we determined classical eye-in-head gain values (eye velocity divided by target velocity) of the respective eye movements. In a first step, all tracking segments were cleaned for saccadic artifacts such as catch-up saccades to analyze the smooth tracking phase solely. Target velocity was determined in two different ways. Since subjects were free to move their eyes, they typically tracked multiple objects during their way through the hallway. In addition, each subject chose his/her own walking speed. Accordingly, the reference velocity (target velocity) had to be determined individually for each subject and each eye-movement trajectory. To this end and as a first approach, we computed the optical flow field from the head-centered video [32]. Target velocity was considered the velocity of the image part relative to the head which was tracked by the subjects’ gaze.

In task IV, subjects had to track one specific object (target) at a time. Thus, target velocity could be easily determined by the temporal derivative of the target position as determined from the head-centered scene. This method was used to compute the gain values. In an additional analysis, we calculated the RMSE (root-mean-square error) of the retinal target velocity. The rationale for choosing the RMSE, just as the gain, is its wide use as a global measure of pursuit performance [33] and its good test–retest reliability [34]. To this end, we analyzed GazeCam videos of the EyeSeeCam, that is, a video sequence obtained from a movable camera which follows the gaze of the subject with a constant latency of about 10 ms [30]. The temporal derivative of the target position within this retinocentric framework served as retinal target velocity. The RMSE corresponds to summing all deviations from this target velocity.

Since most eye-movement parameters during natural vision with free gaze are not normally distributed [35], which also holds true for our data as verified by a Shapiro–Wilk test [36], we used the nonparametric Mann–Whitney *U* test [37] for all statistical analyses. An alpha level of 0.05 was used as threshold for significance. Thus, in task II–IV, we determined the median of each eye movement parameter from each participant separately. Then, the mean and standard deviation of these medians were calculated for the two groups. As an exception, the presence of predefined fixation targets in task I generated a nearly normal distribution of saccadic parameters, which allowed us to calculate the means instead of the medians of each parameter and participant. Additionally, the effect size of each result was computed using the “area under the receiver operating characteristic curve” (AUC) [38]. AUC can be understood as a measure of overlap of two distributions, with separability being minimal at a value of 0.5 and maximal at 0.0 or 1, respectively [39]. The 95 % confidence interval for each effect size was calculated analytically [40].

Finally, as an exploratory post hoc analysis, we correlated (Pearson’s correlation) all eye-movement parameters with the patients’ PANSS score and the corresponding subscores in every task.

## Results

Twenty schizophrenia patients and twenty healthy age- and sex-matched controls participated in the study. All participants had to do four different oculomotor tasks in a natural environment.

### Eye-tracking parameters

We found no significant difference in blink frequency between patients and controls, which otherwise could have compromised the analysis of saccade frequency or fixation duration differently (task I: mean 0.198/s ± 0.408/s vs. 0.135/s ± 0.226/s; *Z* = 0.520; *p* = 0.603; AUC = 0.546 [0.366 0.727]; task II: mean 0.457/s ± 0.314/s vs. 0.301/s ± 0.140/s; *Z* = 1.447; *p* = 0.148; AUC = 0.635 [0.462 0.808] task III: mean 0.629/s ± 0.311/s vs. 0.486/s ± 0.296/s; *Z* = 1.475; *p* = 0.140; AUC = 0.638 [0.465 0.810]).

### Saccades

We determined general saccade features such as frequency, amplitude, and peak velocity for tasks I–III (see Methods for details). During task I, self-initiated saccades between predefined targets showed a significantly larger undershoot in the patient population as compared to healthy controls (Table 2, task I). In contrast, differences in saccade peak velocity between groups did not reach significance. Accordingly, the difference in the main-sequence as represented by the two fitting parameters of the power function only tended to be statistically different (Table 2, task I). Additionally, patients showed a significantly higher saccade frequency as represented by a higher rate of alternating gaze between the predefined targets. Finally, inpatients showed significantly less saccades per second (mean 1.287/s ± 0.456/s) than outpatients (mean 1.806/s ± 0.556/s; *Z* = −2.086; *p* = 0.037; AUC = 0.212 [0.010 0.414]).

**Table 2:** Basic saccadic parameters by task

	Patients Mean (SD)	Controls Mean (SD)	Effect size (AUC)	*U* test	
				*Z* value	*p* value
Task I (stationary targets)					
**Mean saccade amplitude (°)**	**5.53 (1.16)**	**6.57 (1.29)**	**0.305 [0.141 0.469]**	−**2.096**	**0.036**
Mean saccade peak velocity (°/s)	270.0 (50.9)	283.2 (43.3)	0.395 [0.219 0.571]	−1.123	0.262
**Main-sequence fit** ***K*** **value**	**160.0 (37.7)**	**130.7 (58.9)**	**0.690 [0.525 0.855]**	**2.044**	**0.041**
Main-sequence fit *L* value	0.328 (0.13)	0.468 (0.21)	0.338 [0.168 0.507]	−1.745	0.081
**Mean saccade frequency (1/s)**	**1.57 (0.57)**	**1.06 (0.54)**	**0.728 [0.570 0.885]**	**2.448**	**0.014**
Task II (free gaze)					
Median saccade amplitude (°)	2.641 (1.25)	3.014 (0.78)	0.363 [0.190 0.535]	−1.474	0.140
Median saccade peak velocity (°/s)	218.6 (39.2)	218.0 (19.5)	0.488 [0.306 0.669]	−0.122	0.903
Main-sequence fit *K* value	162.37 (23.50)	152.92 (14.78)	0.618 [0.442 0.793]	1.256	0.209
Main-sequence fit *L* value	0.339 (0.11)	0.352 (0.04)	0.550 [0.370 0.730]	−0.527	0.598
**Mean saccade frequency (1/s)**	**0.80 (0.62)**	**1.13 (0.25)**	**0.235 [0.086 0.384]**	−**2.854**	**0.004**
Task III (walking)					
**Median saccade amplitude (°)**	**3.186 (1.41)**	**4.247 (1.22)**	**0.268 [0.111 0.424]**	−**2.512**	**0.012**
Median saccade peak velocity (°/s)	260.1 (56.1)	283.0 (47.5)	0.368 [0.194 0.541]	−1.419	0.156
Main-sequence fit *K* value	190.31 (42.08)	179.02 (29.69)	0.580 [0.402 0.758]	0.852	0.394
Main-sequence fit *L* value	0.307 (0.07)	0.348 (0.06)	0.338 [0.168 0.507]	−1.745	0.081
Mean saccade frequency (1/s)	2.51 (1.59)	2.75 (1.07)	0.423 [0.244 0.601]	−0.826	0.409
Overall					
Median saccade amplitude (°)	2.825 (1.15)	3.175 (0.89)	0.345 [0.178 0.528]	−1.665	0.096
Median saccade peak velocity (°/s)	237.2 (38.5)	239.0 (27.9)	0.473 [0.290 0.658]	−0.285	0.776
**Main-sequence fit** ***K*** **value**	**181.04 (26.64)**	**167.21 (26.63)**	**0.690 [0.525 0.855]**	**2.044**	**0.041**
**Main-sequence fit** ***L*** **value**	**0.312 (0.053)**	**0.349 (0.062)**	**0.318 [0.151 0.484]**	−**1.961**	**0.0499**
Mean saccade frequency (1/s)	1.23 (0.74)	1.32 (0.25)	0.400 [0.223 0.577]	−1.069	0.285
Median saccade peak velocity (°/s) for saccade amplitudes of					
**1°–2°**	**187.1 (** ***n*** **=** **1097)**	**181.4 (** ***n*** **=** **1138)**	**0.535 [0.512 0.559]**	**2.901**	**0.004**
**2°–3°**	**210.1 (** ***n*** **=** **587)**	**203.3 (** ***n*** **=** **633)**	**0.548 [0.516 0.581]**	**2.926**	**0.003**
3°–4°	228.4 (*n* = 399)	220.7 (*n* = 449)	0.518 [0.479 0.557]	0.904	0.366

Median saccade amplitudes, peak velocities, frequency, and main-sequence fit parameters for fixating stationary targets in a freely selectable and self-paced order (task I) both free exploration tasks (task II and task III) and the whole measurement (Overall). In task IV, saccades were not analyzed further. Significant parameters are highlighted in bold

In task II, subjects were free to move their eyes. Here, neither saccade amplitude nor peak velocity showed a significant difference between groups which was also reflected by the fitting parameters of the main-sequence (Table 2, task II). Contrary to task I, saccade frequency was significantly lower in patients as compared to healthy controls.

In task III, we found no difference in saccade frequency, but a significantly smaller median saccade amplitude in patients, which was mainly due to less saccades to the periphery as compared to healthy controls. On the other hand, saccade peak velocity and parameters of the main-sequence were not significantly different in this task (Table 2, task III).

**Fig. 2:**
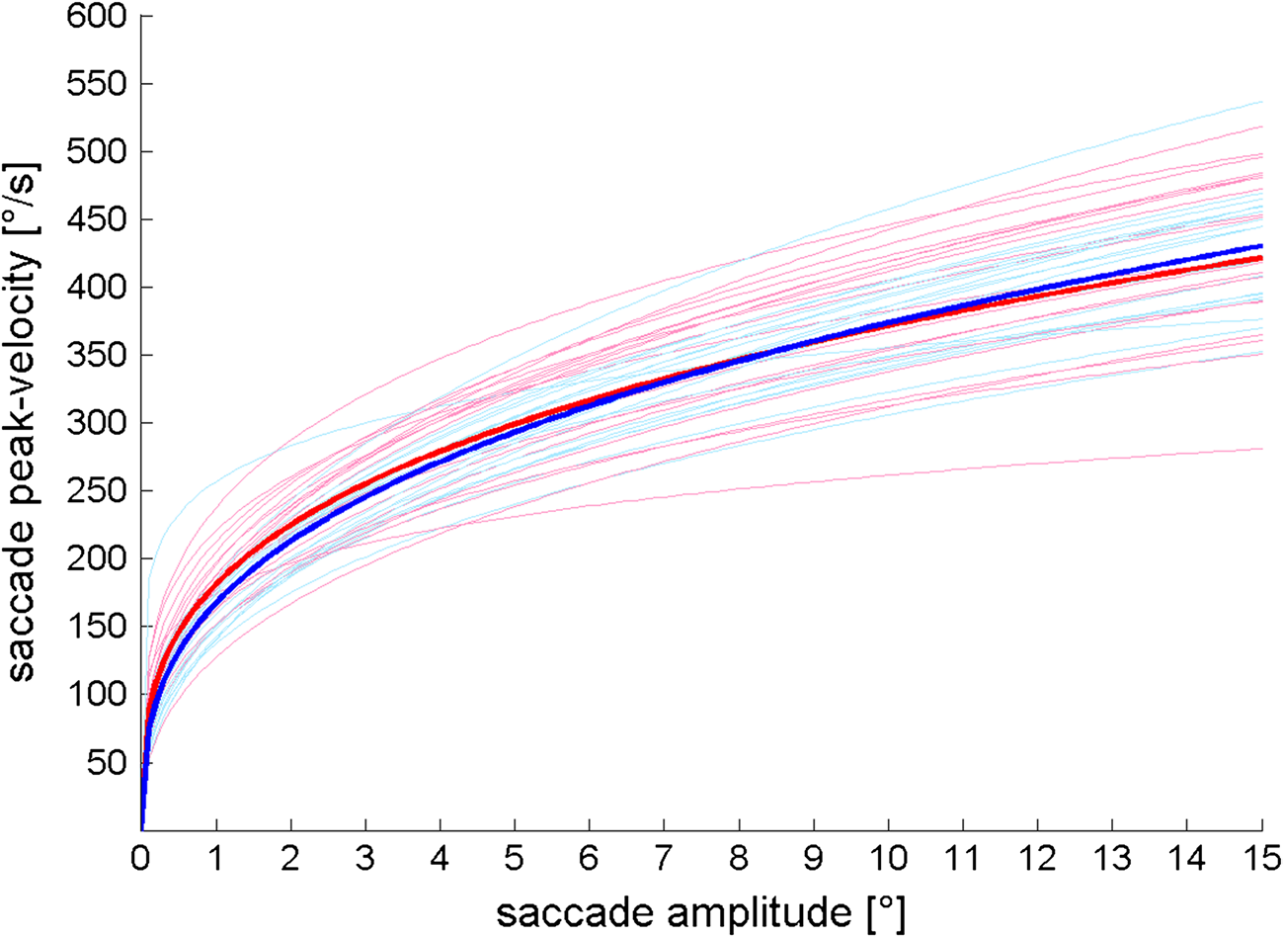
Main-Sequence fit-functions for all participants. Individual data for schizophrenia patients were plotted in *light red*, healthy controls in *light blue*. The mean fit-function of each group is highlighted with a *bold red line* for schizophrenia patients and a *bold blue line* for healthy controls. An initial steeper rise of the mean fit-function in schizophrenia patients is clearly visible for small amplitudes. For higher saccade amplitudes, the slope of the main-sequence mean fit-function became less steep in schizophrenia patients as compared to healthy controls

When analyzing the saccades of the entire measurement without differentiating between certain tasks, which resembles the diversity of saccadic eye movements during everyday life, differences in the main-sequence fit parameters between patients and controls became significant (Table 2, Overall; Fig. 2). Although not statistically different, the quality of the main-sequence fits as represented by the *R* ^2^ values tended to be worse in schizophrenia patients (Overall: mean *R* ^2^ = 0.545 ± 0.185) than in healthy controls (Overall: mean *R* ^2^ = 0.655 ± 0.184; *Z* = −1.91; *p* = 0.057; AUC = 0.323 [0.155 0.490]). The difference of saccade parameters between schizophrenia patients and healthy controls became particularly prominent when analyzing peak velocities of saccades within specific amplitude ranges. Patients showed the most significant differences in saccade peak velocities as compared to controls for small amplitudes, e.g., 1°–2° or 2°–3°. This difference in saccade peak velocities disappeared for saccade amplitudes between 3°–4° and higher amplitude ranges. Across the whole measurement range, neither saccade amplitude nor peak velocity or frequency showed a significant difference between groups.

Some of the eye-movement parameters described here correlated with the patients’ PANSS items (Table 3), notably a negative correlation between mean saccade amplitude in task I and a lack of judgment and insight (*r*(18) = −0.540; *p* = 0.014; Pearson’s correlation) and a positive correlation of median saccade amplitude in task II and motor retardation (*r*(18) = 0.608; *p* = 0.004; Pearson’s correlation). There was no correlation of the saccade parameters with any PANSS item during task III and task IV or with CPZ equivalent dose (all *p* ≥ 0.4).

**Table 3:** Correlations of saccade parameters with patients’ symptom ratings by task

Eye-movement parameter	PANSS item	Pearson’s correlation coefficient (*df* = 18)	*p* value
Task I (stationary targets)			
Mean saccade amplitude (°)	Poor rapport	−0.594	0.006
Mean saccade amplitude (°)	Lack of spontaneity and flow of conversation	−0.538	0.014
Mean saccade amplitude (°)	Stereotyped thinking	−0.481	0.032
Mean saccade amplitude (°)	Lack of judgment and insight	−0.540	0.014
Mean saccade amplitude (°)	PANSS negative	−0.561	0.010
Mean saccade amplitude (°)	PANSS general	−0.482	0.031
Mean saccade amplitude (°)	PANSS total	−0.570	0.009
Mean saccade peak velocity (°/s)	Lack of spontaneity and flow of conversation	−0.452	0.045
Mean saccade peak velocity (°/s)	PANSS negative	−0.511	0.021
Task II (free gaze)			
Mean # saccade (1/s)	Depression	0.480	0.032
Mean # saccade (1/s)	Motor retardation	0.492	0.028
Median saccade amplitude (°)	Motor retardation	0.608	0.004
Median saccade peak velocity (°/s)	Somatic concern	0.491	0.028

Significant correlations of saccade eye-movement parameters with PANSS scores and subscores for task I and task II. Task III and task IV did not show any significant correlations

### Fixation

**Fig. 3:**
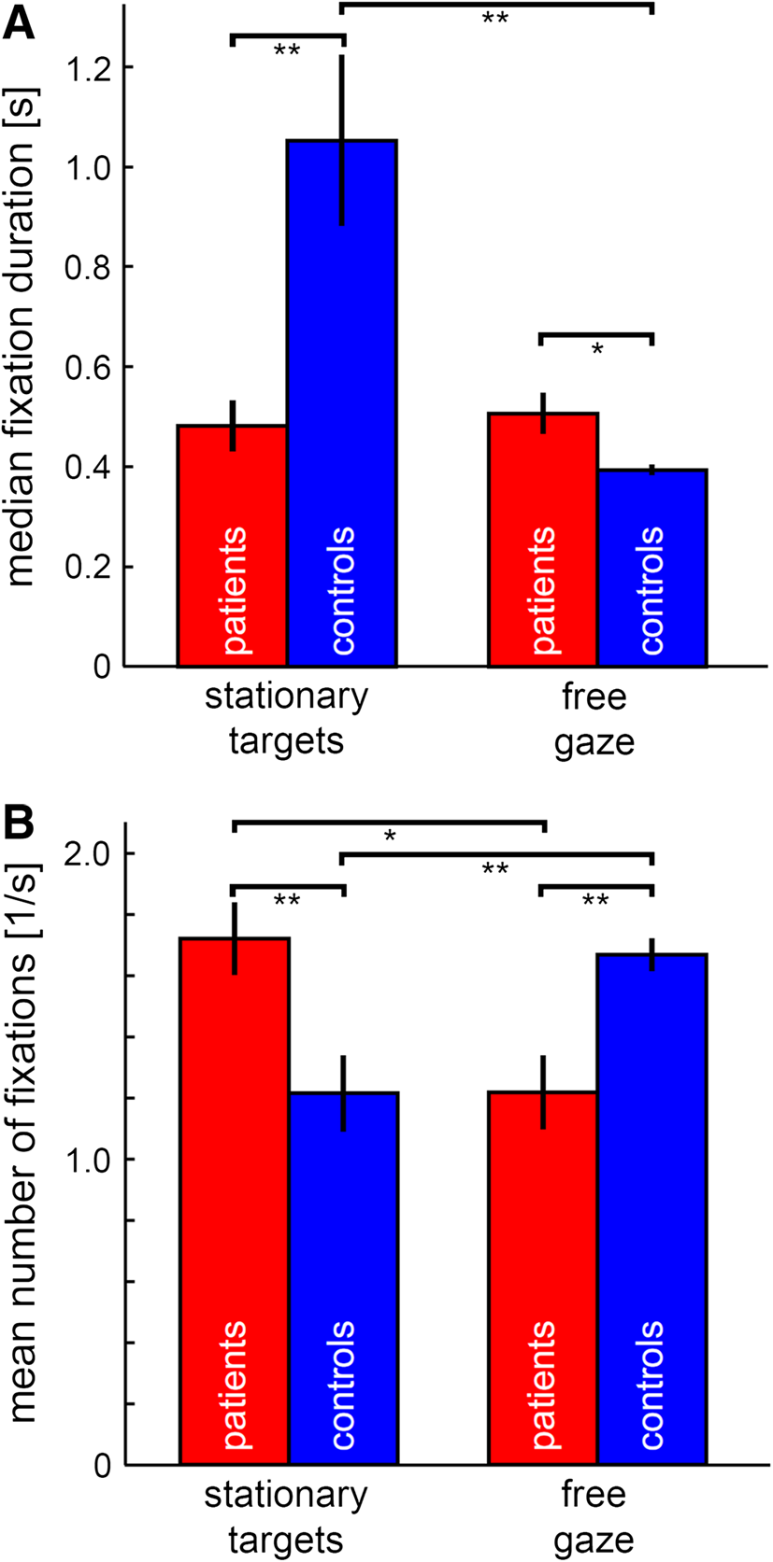
Median fixation duration (**a**) and mean fixation frequency (**b**) during task I (stationary targets) and task II (free gaze). When subjects had to fixate predefined targets in a freely chosen random order, patients fixated significantly shorter and more often than healthy controls. During free gaze it was the other way around. Remarkably, the median fixation duration in patients did not differ significantly between the two tasks, while healthy controls showed a significant modulation of this value. *Vertical black bars* indicate standard error. *Horizontal bars* and the corresponding *stars* indicate significant differences between the two connected values *p < 0.05; **p < 0.01

During free gaze in task II (Fig. 3: free gaze), median fixation duration was significantly longer in patients (0.521 s ± 0.182 s vs. 0.394 s ± 0.045; *Z* = 2.151; *p* = 0.032; *U* test; AUC = 0.700 [0.537 0.863]) and they fixated less often (1.24/s ± 0.51/s) than healthy controls (1.67/s ± 0.23/s; *Z* = −2.962; *p* = 0.003; *U* test; AUC = 0.225 [0.079 0.371]). There was a significant correlation between the PANSS item grandiosity in patients with schizophrenia and median fixation duration during this task (*r*(18) = 0.516; *p* = 0.020; Pearson’s correlation).

Task I, in which subjects had to fixate predefined targets in a freely chosen random order (Fig. 3: stationary targets), showed the exact opposite result. Median fixation duration was significantly shorter in patients (0.481 s ± 0.227 s) as compared to healthy controls (1.053 s ± 0.766 s; *Z* = −3.246; *p* = 0.001; *U* test; AUC = 0.199 [0.060 0.338]), whereas fixation frequency was significantly higher for patients than for controls (1.72/s ± 0.52/s vs. 1.22/s ± 0.56/s; *Z* = 2.61; *p* = 0.009; *U* test; AUC = 0.743 [0.588 0.897]). Furthermore, there was a correlation in schizophrenic patients between anxiety and mean number of fixations per second (*r*(18) = 0.561; *p* = 0.010; Pearson’s correlation) during this task.

 Remarkably, the above finding of an inversion of fixation behavior of patients and controls between free and guided gaze was accompanied by (or due to) the fact that the median fixation duration in patients did not differ significantly between the two tasks (task I: 0.481 s ± 0.227 s vs. task II: 0.521 s ± 0.182; *Z* = −1.069; *p* = 0.285; *U* test; AUC = 0.400 [0.223 0.577]), while healthy controls showed a significant modulation of this value (task I: 1.053 s ± 0.766 s vs. task II: 0.394 s ± 0.045; *Z* = 3.855; *p* = 0.0001; *U* test; AUC = 0.858 [0.738 0.977]). There were no qualitative or statistical differences in the fixation results, when eye traces were either cleaned for blink artifacts or not.

There was no correlation of any of the fixation parameters with CPZ equivalent dose (all *p* ≥ 0.096).

### Tracking eye movements

In task III, the subjects looked at self-chosen targets (e.g., posters at the wall or chairs) while walking through the hallway. This oculomotor behavior induces visual tracking. We analyzed such tracking periods by evaluating the gain of the eye movement. Surprisingly, we found no significant difference in gain between patients (1.316 ± 0.386) and healthy controls (1.246 ± 0.248; *Z* = 0.500; *p* = 0.617; *U* test; AUC = 0.548 [0.367 0.728]) during such spontaneous tracking.

The active tracking of stationary targets on the ground during task IV was also quantified by computing the oculomotor gain values which, again, did not show a significant difference between groups (mean 0.862 ± 0.302 vs. 0.902 ± 0.213; *Z* = −0.448; *p* = 0.654; *U* test; AUC = 0.470 [0.341 0.599]). Yet, the mean RMSE of the foveal velocity, as measure of tracking precision, was significantly higher in patients (23.52°/s ± 10.42°/s) than in healthy controls (16.98°/s ± 6.00°/s; *Z* = 2.385; *p* = 0.017; *U* test; AUC = 0.671 [0.541 0.801]) during this task.

There was no correlation of the parameters during tracking eye movements with any PANSS item or with CPZ equivalent dose (all *p* ≥ 0.136).

## Discussion

In this study, we investigated oculomotor behavior in schizophrenia patients and healthy controls during natural behavior, where participants could freely move their eyes, head, and body. For specific oculomotor parameters, such as fixation duration and frequency, we found significant differences between the groups. Some of these differences resembled those reported under laboratory conditions, such as decreased exploratory eye movements [8, 9], while others (e.g., tracking eye movement gain) seem to reach normal performance during natural vision. This might be a characteristic feature of eye-movement behavior in real-life environments which possibly trigger as of yet unknown compensatory mechanisms.

### Saccades

In task I, subjects had to successively fixate predefined targets in a self-chosen serial order. Similar to results obtained from experiments in a laboratory environment [6, 7], we found a systematic saccadic undershoot in schizophrenia patients. Other studies performed in laboratory environments did not find shortened saccade amplitudes [41] or reported even an overshoot [42]. A possible explanation for these seemingly contradictory results could be the discrete ranges of saccadic amplitudes employed in the different studies. While Schmid-Burgk and colleagues [6, 7] analyzed a variety of saccadic amplitudes, Levin et al. [41] did not analyze each amplitude range separately for its accuracy. This latter approach might have concealed an undershoot for certain saccade amplitudes. When considering the full range of amplitudes, we found no significant difference of peak velocity between patients and controls. When splitting analysis by saccade amplitudes, however, our results during free gaze showed the most prominent differences in saccade peak velocity between patients and controls for small amplitudes (i.e., 1°–3°). This latter amplitude range is most common during natural vision [35] but rarely examined in laboratory settings. Hence, our present results, at least to some extent, reconcile the apparently conflicting findings in the literature.

The main-sequence of saccades only showed significant differences between patients and controls when comparing all saccades during all tasks. The slightly worse *R* ^2^ values in schizophrenia patients could mainly be attributed to the higher variability of saccade parameters within the patient cohort (see standard deviations of saccade amplitudes and peak velocities in Table 2) due to the heterogeneity of the disease. The fit parameters of the power functions indicated an initially steeper slope of functions obtained from patients, which levels out and eventually drops below the level of healthy controls. This functional characteristic might be further evidence that the examined saccade amplitude ranges play a crucial role when studying saccades of schizophrenic patients.

Our results indicate a general impairment of visually guided saccades in schizophrenia patients, which has also been deduced from fMRI findings showing a decreased activation in supplementary and frontal eye fields during saccades [43, 44]. Other studies suggested an impaired prediction of the sensory consequences of one’s own actions [45] and deficient self-monitoring in schizophrenia patients [46], which might result in an incorrect saccade planning. This is supported by studies indicating a generally impaired efference copy mechanism in patients with schizophrenia [47–49]. The negative correlation of the saccade amplitude with the severity of symptoms in our study is in line with these findings and offers an explanation for the observed saccadic undershoot during task I in schizophrenia. In our current study, however, differences in saccade parameters between patients and controls largely depended on the amplitude range under consideration. This amplitude-dependent impairment and its normalization in certain ranges demonstrate the ability of patients to compensate, at least partially, for oculomotor inaccuracies during free natural vision.

### Fixation

Oculomotor parameters such as fixation duration and fixation frequency were differently influenced by the behavioral task or the presence or absence of predefined targets. During free gaze and without any additional behavioral task, patients fixated significantly longer and less often than healthy controls. This is in line with a recent study by Egaña et al. [9], investigating free viewing of natural images in the laboratory. In our study, median fixation duration was correlated with the PANSS item grandiosity in schizophrenia patients, indicating that some patients might have drifted off in their imagination during this task. During the fixation of given targets and the instruction to look at them in a self-chosen and self-paced order, however, patients leaped from target to target more than twice as fast as compared to healthy controls. This performance was significantly correlated with anxiety in schizophrenia patients leading to even shorter fixation durations and more fixations per second the more anxious a patient was. However, the observed differences between the tasks were mainly due to a task-specific change in the median fixation duration of healthy controls, while schizophrenia patients fixated almost equally long in both tasks. These results could be based on different bottom-up and top-down processing of visual and non-visual information in schizophrenia patients and healthy controls. It has been argued, for example, that the deployment of attention, for which gaze allocation is a proxy [50, 51], is altered in patients with schizophrenia. Various studies have shown a higher distractibility [52] or the inability to focus attention on salient cues [53] in schizophrenia patients. Yet, the lack of difference in fixation duration during task I and task II in schizophrenia patients of our study implies a more subtle influence of attention and task demands on patients as compared to controls. This suggests that alteration of task performance is differently modulated in patients with schizophrenia and that top-down influences might be less influential for their behavior as compared to healthy controls.

Overall eye-movement patterns of schizophrenia patients showed less exploratory behavior such as saccades to the periphery. This result might be indicative for a generally lower interest of patients in exploring their environment. Earlier studies showed similarly decreased exploratory eye movements in schizophrenia patients in laboratory settings [8, 9] or during unfamiliar tasks in a real-life scenario [10]. We show that this finding is also valid during natural vision in everyday life and therefore might influence perception as a whole in schizophrenia patients.

### Tracking eye movements

The analysis of the visual tracking of a stationary target on the ground during self-motion revealed an unexpected result. Although related to smooth-pursuit eye movements (i.e., keeping a visually moving object stationary on the retina), we could not find the typical reduced tracking gain, which has been reported for schizophrenia patients and even their first-degree relatives under laboratory conditions [1–4, 6]. Instead, patients and controls revealed high gain tracking of stationary targets and freely chosen objects. Active tracking of optic flow elements with a gain of almost 1.0 has previously been described under laboratory conditions for healthy subjects [14]. Our current findings indicate that patients might be able to partly compensate for their poor tracking performance during smooth pursuit in the laboratory, e.g., using additional sensory cues (optic flow, vestibular signals) when tracking a target in a real-world environment. This view is supported by Holzman [54], who identified the main source of poor tracking performance in schizophrenia patients as a deficit in velocity sensitivity. In his study, the velocity discrimination of patients got worse when additional non-velocity stimulus cues were eliminated and subjects were forced to rely solely on velocity cues. In natural behavior, several sensory and motor signals interact (e.g., a combination of pursuit and vergence eye movements), which might aid the visual and oculomotor system during target tracking. Additionally, in our paradigm, head movements may have compensated for the otherwise impaired tracking gain. This idea is supported by a recent study which showed abnormal eye–head coordination in schizophrenia patients expressed by an uneconomic over-performance of head movements [55]. Finally, a generally higher demand during natural tasks might have influenced the tracking performance of schizophrenic patients. Shagass and colleagues [56] showed that smooth-pursuit gain in the laboratory improved significantly when the patients had to read numbers shown on the tracking target. The authors argued that the improved gain was due to an increased attentional load, which might also apply to tracking eye movements in natural environments.

Contrary to the gain-tracking performance, the RMSEs of the foveal velocity differed significantly between schizophrenia patients and healthy controls. This result suggests a generally more imprecise tracking with numerous small deviations from an optimal tracking behavior in patients with schizophrenia.

Since re-inviting the same cohort of patients to laboratory measurements was not feasible, we compared the real-world data to common findings from laboratory data reported in the literature. It is self-evident that there is no one-to-one mapping between such tasks.

For example, smooth-pursuit eye movements with fixed head in the laboratory and tracking (eye)movements in the real world serve the same purpose: keep a visually moving object stationary on the retina. However, in the former case only, the eyes are moving, whereas in the latter, eye, head, and possibly body contribute. Hence, the real-world situation requires a higher level of integration, but also offers mechanisms for compensation. To further investigate the differences in tracking performance and the possible contribution of head movements and additional sensory signals, a future study could analyze the tracking of a thrown object while participants are not moving. This type of experiment might be more comparable to smooth pursuit in the laboratory and might reduce the gap between the reported reduced gain in the literature and the real-world data in our study.

Another challenge for comparing data between studies—and even for between-subject designs in the same study—is the heterogeneity of schizophrenia, which is further amplified by potential effects of medication. These limitations notwithstanding, any differences between our results and studies performed in the laboratory may suggest the influence of as of yet unknown distinct or additional mechanisms to eye movements of schizophrenia patients in natural environments, which would point toward new research objectives of future studies and will help to complement the overall picture of this disease.

Schizophrenia patients perform worse in a variety of visual motion tasks, such as discrimination of velocity [57] and motion direction [20], localization and visual backward masking tasks [58]. This may be caused by a dysfunction in areas of the visual motion system, i.e., among others in human middle temporal area (MT) and MST [20, 59], respectively. A dysfunction in those areas in schizophrenia patients could also contribute to the more noisy tracking behavior in our study. Studies in non-human primates implicate that another area of the parietal cortex, the ventral intraparietal area (VIP), is critically involved in the encoding of self-motion [23, 60, 61] and smooth-pursuit eye movements by guiding and coordinating smooth eye and head movements within near-extrapersonal space [62–64]. A functional equivalent of macaque VIP has been identified in human [22]. Accordingly, human area VIP might also play a crucial role in the observed eye-movement dysfunction in schizophrenia patients. This view is supported by Chen et al. [20] who showed a global, but not local, motion processing deficit in patients with schizophrenia. The contribution of multisensory areas like area VIP [65] to the eye-movement behavior in schizophrenia patients might have been hidden in most previously conducted laboratory studies and becomes especially interesting in natural contexts by providing and combining additional sensory information. Hence, further investigations of a functional impairment of the areas within the parietal cortex of schizophrenia patients are needed to better understand the observed eye-movement deviations from healthy controls during natural behavior.

In conclusion, the study of eye movements in natural environments showed differences in basic eye-movement parameters between schizophrenia patients and healthy controls during simple everyday tasks, which were strongly modulated by the task demands. Furthermore, our data suggest that patients can overcome some oculomotor impairments, which become obvious in laboratory studies (e.g., reduced gain during tracking eye movements), by as yet unknown compensatory mechanisms or strategies. These might include an improvement in performance due to higher task engagement and additional sensory input (optic flow, vestibular signals) during natural tasks as well as the possibility to perform unrestricted head movements. Being aware of the multitude of differences between our real-world tasks and typical laboratory measurements, our results provide a first step toward analyzing real-world oculomotor behavior in schizophrenia. Teasing apart the sources of differences and commonalities between laboratory results and real-world data will be an important issue for future research. In any case, our results underline the need to complement laboratory experiments with real-word data (and vice versa) in order to achieve a complete picture of oculomotor dysfunctions in schizophrenia and their implications for patients’ activities of daily living.
